# Supplementary material for: Widespread extracellular electron transfer pathways for charging microbial cytochrome OmcS nanowires via periplasmic cytochromes PpcABCDE
Source: Nat Commun. 2024 Mar 20;15:2434. doi: 10.1038/s41467-024-46192-0 (PMC10954620; doi:10.1038/s41467-024-46192-0)
Supplement: Supplementary file 1 — Supplementary Information [file 41467_2024_46192_MOESM1_ESM.pdf]

Supplementary Tables and Figures

This file contains Supplementary Figures 1-2 and Supplementary Tables 1-2.

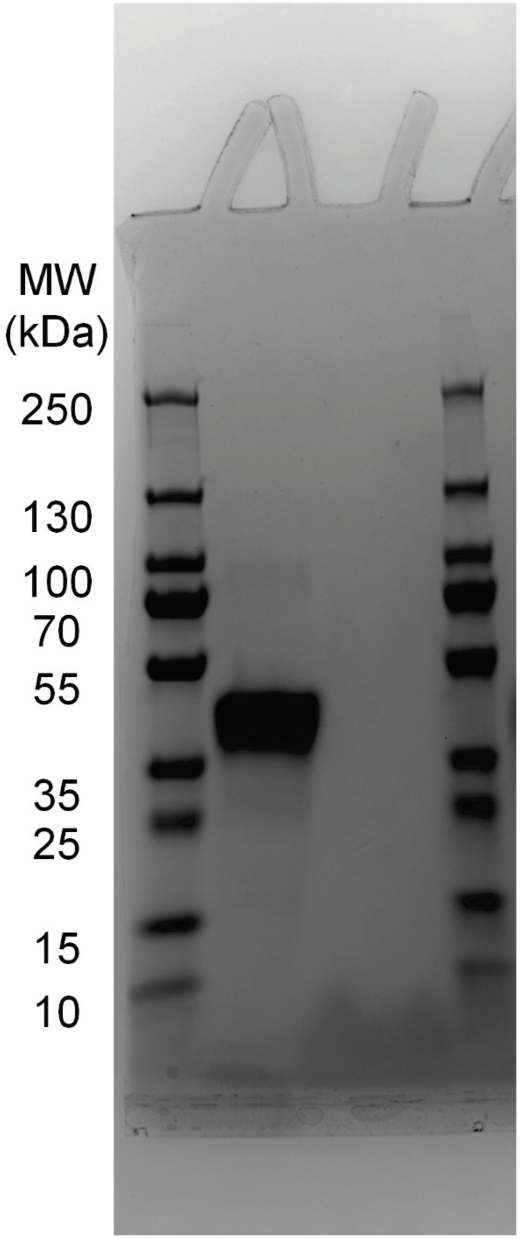

Supplementary Fig. 1. Purification and characterization of cytochrome OmcS nanowires. Full length Coomassie-stained SDS-PAGE gel of purified OmcS nanowires showing sample purity.

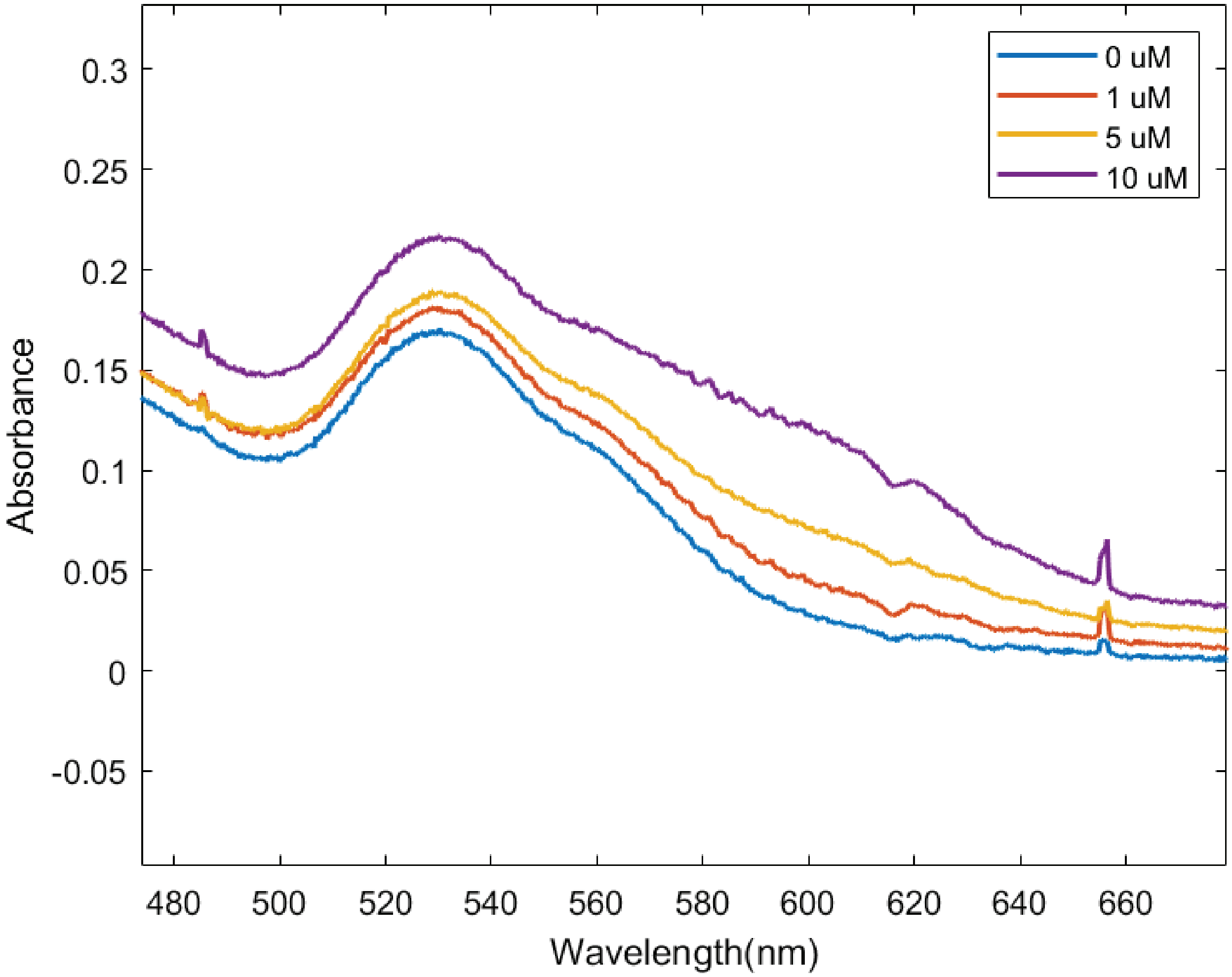

Supplementary Fig. 2. Optimization of mediator concentration to maximize SEC signal and minimize signal interference. The absorbance of 37µM OmcS nanowires with the increasing concentration of mediators at 0, 1, 5, and 10 µM or 0, 0.5, 2.5, and 5% ratio of mediators to hemes in the sample. The mediators' spectrum is strongest around 600 nm when oxidized. The spectrum around the α-band remains undistorted below 2.5%. Therefore, we chose a mediator ratio in 1-2% to avoid the signal interference.

Supplementary Table 1 – ^1H chemical shift values of the heme substituents probed in this work in the absence and in the presence of OmcS (1:4 PpcA-E: OmcS heme ratio). N.D stands for a chemical shift that was not observable.

Heme	Heme substituent	Chemical shift in the absence of OmcS					Chemical shift in the presence of OmcS				
		PpcA	PpcB	PpcC	PpcD	PpcE	PpcA	PpcB	PpcC	PpcD	PpcE
I	2^1CH_3	17.80	14.77	16.75	26.02	13.31	17.80	14.78	16.84	26.03	13.32
	7^1CH_3	10.50	13.78	11.27	N.D	14.04	10.50	13.77	11.17	N.D	14.03
	12^1CH_3	21.21	17.63	22.92	21.38	14.33	21.21	17.65	23.03	21.38	14.35
	18^1CH_3	15.66	17.54	19.08	25.07	20.11	15.66	17.52	18.97	25.07	20.10
III	2^1CH_3	12.09	13.34	11.73	10.15	13.37	12.08	13.34	11.72	10.15	13.37
	7^1CH_3	18.07	15.95	19.95	15.69	12.32	18.07	15.95	19.95	15.69	12.32
	12^1CH_3	13.18	17.32	12.14	16.15	19.95	13.17	17.32	12.13	16.15	19.95
	18^1CH_3	N.D	N.D	N.D	N.D	N.D	N.D	N.D	N.D	N.D	N.D
IV	2^1CH_3	14.71	15.48	20.26	12.69	15.16	14.71	15.48	20.39	12.69	15.16
	7^1CH_3	10.63	9.54	N.D	14.34	9.38	10.62	9.54	N.D	14.34	9.39
	12^1CH_3	19.06	19.72	24.27	13.43	18.83	19.10	19.73	24.31	13.41	18.84
	18^1CH_3	14.80	14.22	10.73	16.97	14.62	14.80	14.22	10.63	16.97	14.62

Supplementary Table 2 – ^{1}H and ^{15}N chemical shift values of PpcA's backbone in the absence and in the presence of OmcS (1:4 PpcA: OmcS heme ratio).

Amino acid	Chemical shift in the absence OmcS		Chemical shift in the presence OmcS	
	^{1}H	^{15}N	^{1}H	^{15}N
ASP^{3}	9.65	120.57	9.66	120.58
ILE^{4}	10.29	124.29	10.28	124.34
VAL^{5}	9.43	129.64	9.43	129.66
LEU^{6}	9.71	132.88	9.71	132.89
LYS^{7}	8.51	125.05	8.51	125.05
ALA^{8}	9.95	123.7	9.92	123.58
LYS^{9}	10.05	125.24	10.05	125.25
ASN^{10}	9.76	114.92	9.76	114.92
GLY^{11}	9.62	113.71	9.63	113.72
ASP^{12}	10.37	133.49	10.36	133.48
VAL^{13}	10.90	123.15	10.92	123.15
LYS^{14}	9.94	129.76	9.93	129.69
PHE^{15}	9.91	125.73	9.91	125.64
HIS^{17}	11.64	138.52	11.64	138.54
LYS^{18}	13.16	126.43	13.20	126.47
ALA^{19}	11.06	124.56	11.07	124.56
HIS^{20}	12.19	118.58	12.21	118.6
GLN^{21}	9.92	121.62	9.92	121.63
LYS^{22}	8.13	117.15	8.13	117.14
ALA^{23}	8.09	119.77	8.09	119.78
VAL^{24}	8.44	119	8.44	119.02
ASP^{26}	7.21	118.37	7.21	118.38
CYS^{27}	7.52	124.65	7.51	124.65
LYS^{28}	8.39	115.15	8.39	115.17
LYS^{29}	7.5	118.58	7.5	118.58
CYS^{30}	6.68	111.11	6.68	111.14
HIS^{31}	12.03	122.48	12.04	122.51
GLU^{32}	10.51	128.75	10.51	128.75
LYS^{33}	10.36	117.21	10.37	117.22
GLY^{34}	9.06	109.06	9.07	109.06
LYS^{37}	7.5	118.22	7.49	118.26
ILE^{38}	10	128.47	10.01	128.47
GLU^{39}	9.38	131.36	9.38	131.37
GLY^{40}	9.06	113.18	9.06	113.17
PHE^{41}	7.71	121.64	7.71	121.67
LYS^{43}	7.39	121.42	7.39	121.4
GLU^{44}	8.87	117.61	8.87	117.64
MET^{45}	8.27	119.24	8.27	119.26
ALA^{46}	7.79	121	7.8	121.06
HIS^{47}	11.83	118.6	11.85	118.63
GLY^{48}	8.67	112.44	8.68	112.49
GLY^{50}	7.77	104.47	7.77	104.48
LYS^{52}	7.76	121.75	7.75	121.73
GLY^{53}	9.39	104.86	9.39	104.86

Amino acid	Chemical shift in the absence OmcS		Chemical shift in the presence OmcS	
	^{1}H	^{15}N	^{1}H	^{15}N
**CYS^{54}**	6.25	118.95	6.25	118.99
**HIS^{55}**	10.16	119.14	10.16	119.13
**GLU^{56}**	9.91	119.18	9.91	119.19
**GLU^{57}**	8.39	121.05	8.38	121.05
**MET^{58}**	9.33	114.91	9.34	114.91
**LYS^{59}**	8.19	115.28	8.19	115.27
**LYS^{60}**	8.27	118.32	8.26	118.3
**GLY^{61}**	6.13	102.39	6.12	102.39
**THR^{63}**	8.81	111.44	8.81	111.47
**LYS^{64}**	8.09	123.7	8.09	123.69
**CYS^{65}**	6.76	116.62	6.77	116.61
**GLY^{66}**	8.39	102.88	8.39	102.88
**GLU^{67}**	7.05	115.94	7.05	115.96
**CYS^{68}**	6.61	113.37	6.6	113.4
**HIS^{69}**	11.59	121.24	11.58	121.24
**LYS^{70}**	10.37	127.37	10.38	127.39
**LYS^{71}**	9.11	129.98	9.11	130
